# CXCL9 may serve as a potential biomarker for primary Sjögren’s syndrome with extra-glandular manifestations

**DOI:** 10.1186/s13075-023-03229-x

**Published:** 2024-01-17

**Authors:** Jingwei Hong, Hui Cheng, Ping Wang, Yanzhi Wu, Saisai Lu, Yan Zhou, Xiao bing Wang, Xiaofang Zhu

**Affiliations:** 1 Rheumatology Department, First Affiliated Hospital of Wenzhou Medical University, Nanbai Xiang Street, Ouhai District, Wenzhou, China; 2 Department of Rheumatology and Immunology, Shanghai Changzheng Hospital, Second Affiliated Hospital of Naval Medical University, Shanghai, China

**Keywords:** Primary Sjögren’s syndrome, Extra-glandular manifestations, CXCL9, RNA-sequencing, ESSDAI

## Abstract

**Background:**

Primary Sjögren's syndrome (pSS) is an autoimmune condition that causes harm to exocrine glands and also has extra-glandular manifestations (EGM). pSS patients with EGM have a worse prognosis than those with only sicca symptoms. Previous studies have shown that the minor salivary glands (MSG) of pSS patients exhibit a unique profile of cytokines and chemokines compared to healthy controls. However, there is a lack of research comparing pSS with EGM (pSS-EGM) and pSS without EGM (pSS-non-EGM). This study aims to explore potential biomarkers associated with pSS, particularly pSS with EGM.

**Methods:**

By utilizing RNA sequencing, we conducted an analysis on the gene expression profiles of MSG in 63 patients diagnosed with pSS, as well as 12 non-pSS individuals. Furthermore, we also investigated the MSG of pSS patients, both with and without EGM. Through bioinformatics analysis, we identified genes with differential expression (DEGs) and determined the core hub genes using PPI network. We then analyzed the top 20 DEGs and their correlation with the patients' clinical characteristics, and validated our findings using peripheral blood plasma.

**Results:**

A total of 725 differentially expressed genes (DEGs) were identified in the comparison between pSS and non-pSS groups, and 727 DEGs were observed between pSS-EGM and pSS-non-EGM. It is noteworthy that the expression levels of *CXCL9* were higher in both pSS patients and pSS-EGM when compared to the control group. Taking into consideration the significance of the top 20 DEGs in relation to clinical parameters and the central hub genes, we ultimately chose *CXCL9*. In comparison to the non-pSS group, pSS patients exhibited notably greater expression of the *CXCL9* gene in the MSG, as well as higher levels of CXCL9 protein in their plasma (*p* < 0.001). Furthermore, the expression of the *CXCL9* gene and levels of CXCL9 protein were notably higher in pSS patients accompanied by EGM and those with SSA antibodies. Additionally, a correlation was found between the expression of the *CXCL9* gene and the EULAR Sjogren’s Syndrome Disease Activity Index (ESSDAI), as well as with immunoglobulin G (IgG) levels and erythrocyte sedimentation rate (ESR). Meanwhile, the protein levels of CXCL9 were found to be correlated with IgG levels and ESSDAI.

**Conclusion:**

CXCL9 proves to be a valuable biomarker in pSS, specifically due to its strong ability to differentiate between pSS patients with EGM and those without EGM. There is a significant correlation between CXCL9 and various clinical parameters both at the gene and protein level. Therefore, CXCL9 could be a potential target for future treatment of pSS.

**Supplementary Information:**

The online version contains supplementary material available at 10.1186/s13075-023-03229-x.

## Introduction

Primary Sjögren's syndrome (pSS) is an autoimmune condition that usually starts with gradual sicca symptoms [1, 2], but approximately 40% of patients may have extra-glandular manifestations (EGM) [3]. These EGM can affect different areas of the body, such as joints, skin, lungs, kidneys, and nervous system, and may range in severity.

They not only significantly impact a person's overall health, but also increase the burden of the disease, in addition to causing dryness [4, 5]. EGM in patients with pSS affects the prognosis of patients [4, 6, 7].

The precise cause of pSS is not currently fully understood. However, lymphocytes have traditionally been believed to play a key role in its development [8]. Chemokines, which are small proteins, attract immune cells and interact with chemokine receptors to facilitate their movement and differentiation [9]. Research has indicated that the levels of CXCL13 increase before the disease becomes clinically evident. This overexpression of CXCL13 promotes the formation of abnormal lymphoid tissues primarily composed of B lymphocytes in pSS. Additionally, the use of anti-CXCL13 antibodies has shown effectiveness in managing pSS [10, 11]. Furthermore, studies have found that CXCL9, CXCL10, and CXCL11 are upregulated on the corneal and conjunctival epithelium in response to desiccating stress in mice and in patients with dry eye [12, 13]. In 2023, Zhang et al. discovered that the CXCL9, 10, 11/CXCR3 axis, which activates G protein-coupled receptor kinase 2, is involved in regulating the migration of T lymphocytes, contributing to the development of pSS [14]. Inhibiting the CXCL9, 10, 11/CXCR3 axis or G protein-coupled receptor kinase 2 can reduce the migration of T lymphocytes [14].

A key factor in diagnosing pSS is the use of a minor salivary gland (MSG) biopsy [15, 16]. Pathologists analyze the minor salivary gland, and the existence of one or more lymphocytic foci with over 50 lymphocytes per 4 mm^2^ is considered highly indicative of pSS [16, 17]. This is a critical diagnostic criterion for the disease. Additionally, previous research [18–20], along with our own [21, 22], has shown that patients with pSS have a distinct gene-expression signature in their MSG that sets them apart from healthy individuals. Most of these DEGs are upregulated, such as type I interferon, chemokine *CXCL9*, *CXCL10*, and *CXCL13* [23–27].

While most studies focus on patients with primary Sjögren's syndrome (pSS) and non-pSS patients, as well as healthy individuals, limited research exists on patients with pSS associated with EGM and pSS without EGM. In this study, we examined the clinical traits and gene expression of minor salivary gland (MSG) and plasma samples. We compared these samples not only between individuals diagnosed with pSS and those without the diagnosis but also between pSS-EGM and pSS-non-EGM patients, aiming to identify potential biomarkers through the comparison of various indicators.

## Materials and methods

### Patients and sample collection

Between January 2020 and August 2021, a group of 63 pSS patients and 12 non-pSS subjects were enrolled at the First Affiliated Hospital of Wenzhou Medical University in Zhejiang, China. Based on the 2012 ACR criteria or the 2016 (ACR)/EULAR classification criteria, pSS was diagnosed in this study [28, 29]. The non-pSS subjects were mainly from the physical examination center of our hospital. This group of patients was treated in our Rheumatology and Immunology department because they were positive for antibodies or experienced discomfort with dry mouth or dry eyes. Eventually, the diagnosis of pSS was ruled out based on negative antibodies, MSG biopsy, ophthalmic KCS examination, and saliva flow rate testing. MSG biopsy was performed on all patients for diagnostic purposes.

The inclusion criteria for this study were as follows: ① Willing and voluntary participation of all participants. ② Signing of an informed consent form by all participants. ③ Age range between 18 and 70 years for participants. The exclusion criteria were: ① Patients who had received treatment (including glucocorticoids, immunosuppressants, or biological agents). ② Patients who were currently pregnant or lactating. ③ Patients with another known connective tissue disease or overlap syndrome. ④ Patients with acquired immunodeficiency. ⑤ Patients with a history of diagnosed malignant tumors. ⑥ Patients with recent infections.

At the same time as the MSG biopsies, we collected salivary glands and plasma samples from all participants to analyze the results. In addition, plasma samples were collected from 10 patients with systemic lupus erythematosus (SLE) (Table S1) and 20 healthy individuals. To avoid confounding effects, collect all data, including clinical information and EULAR Sjögren's Syndrome Disease Activity Index (ESSDAI) scores [30, 31], prior to administering any systemic immunosuppressant or glucocorticoid medications. Systemic involvement was defined according to the ESSDAI [31]. The data gathered encompassed patient age, dry eyes or mouth symptoms, test results for ocular examinations, and symptoms of extra-glandular involvement. Other documented variables included active joint involvement, interstitial lung disease (ILD) [32], skin vasculitis, peripheral neurological involvement, and renal involvement characterized by persistent proteinuria, tubular acidosis, interstitial nephritis, or glomerulonephritis. Additionally, laboratory investigations, including anti-nuclear antibodies (ANA), anti-SSA antibody (SSA), anti-SSB antibody (SSB), rheumatoid factor (RF), levels of immunoglobulins, and complement component levels (C3 and C4), etc., were extracted from the patient’s clinical record.

The study (#16024) was approved by the ethics committee of the First Affiliated Hospital of Wenzhou Medical University.

### Extraction of RNA, preparation of cDNA libraries, and sequencing

Gene expression was investigated in this study using RNA sequencing. We extracted total RNA from frozen salivary gland samples by utilizing TRIzol Reagent. The RNA purity was determined by utilizing a Nano Photometer spectrophotometer. To evaluate RNA quality, we employed a Bioanalyzer 2100 system with an RNA 6000 Nano kit. To generate sequencing libraries for Illumina, the NEBNext Ultra RNA Library Prep Kit was utilized using a 3 mg RNA sample. We utilized the Illumina HiSeq platform for sequencing. To improve data quality, we used TrimGalore software and Cutadapt adapters to trim raw reads and filter low-quality reads. With FastQC software, we also generated quality-control reports for sequence reads. DESeq2 was used to normalize read count files after aligning them with the human reference genome “hg38”.

### Bioinformatics analysis

The DEseq2 packages were utilized to identify the differentially expressed genes (DEGs). The cluster profiler package in R was used to examine the annotation of Gene Ontology (GO) and Kyoto Encyclopedia of Genes and Genomes (KEGG) pathway [33]. A threshold for significance was established with a criterion of adjusted *p*-value < 0.05. We evaluated the interactive connections and protein–protein interaction (PPI) networks of the shared DEGs by utilizing the STRING database [34]. The biological network of important DEGs [25] was created and visualized using Cytoscape software [35].

### Enzyme-linked immunosorbent assay

The plasma levels of C-X-C motif chemokine ligand 9 (CXCL9) and soluble C-X-C motif chemokine receptor 3 (CXCR3) were measured in pSS patients, SLE patients, non-pSS subjects, and healthy controls using the Human CXCL9/MIG ELISA Kit from MULTI SCIENCES (Hangzhou, Zhejiang, China) and the Human Chemokine C-X-C-Motif Receptor 3 (CXCR3) ELISA Kit from Jianglai Biological (Shanghai, China), following the product guides. To measure the color produced by 3,3′,5,5′-tetramethylbenzidine (BD Biosciences, United States), the ELISA plate reader was used to record the absorbance at 450 nm. Three tests were conducted on each sample.

### Statistical analysis

We analyzed the data using R version 4.2.0. The mean and standard error (SE) were calculated using a two-sample t-test. DEGs were identified based on a log2 fold change that was either less than -1 or greater than 1, along with an adjusted *p*-value below 0.001. Functional enrichment analysis was performed using the clusterprofiler package. For data that followed a normal distribution, the Student’s t-test was employed, whereas the Mann–Whitney U test was utilized for variables that did not exhibit normality. Categorical variables were compared using chi-square tests. Spearman's correlation analysis was utilized to quantify the connections between gene expression and clinical parameters. A *p*-value less than 0.05 was used to define statistical significance.

## Results

### Patient characteristics

In this study, we compared the characteristics of patients with pSS and non-pSS. Table 1 summarizes the characteristics of 63 pSS patients and 12 non-pSS subjects. Our results show that a greater proportion of pSS patients test positive for ANA, anti-SSB, and anti-SSA antibodies (all* p*-values < 0.05) compared to those without pSS. Nevertheless, neither group showed any significant differences in age or gender.

**Table 1:** Clinical characteristics of patients with pSS and non-pSS

Characteristics	pSS	Non-pSS	*P*-value
N	63	12	
Age, years, mean (range)	50.85 ± 15.90	48.33 ± 12.03	0.542
Female, n (%)	55/63 (87.30)	11/12 (91.67)	1
ANA-positive (%)	42/63(66.67)	1/12 (8.33)	< 0.001
Anti-SSA-positive (%)	49/63(77.78)	1/12 (8.33)	< 0.001
Anti-SSB-positive (%)	28/63 (44.44)	0/12 (0.00)	0.010

Data are expressed as mean ± SD or n (%)

*ANA* Antinuclear Antibody, *SSA* Anti-SSA antibody, *SSB* Anti-SSB antibody

We further analyzed the characteristics of pSS patients and categorized them into two groups: those with EGM and those without EGM. Table 2 presents the characteristics of these two groups. Compared to pSS-non-EGM patients, pSS patients with EGM have higher levels of ANA, RF, ESR, IgG and ESSDAI (all *p*-values < 0.05). Nevertheless, there was no significant difference in anti-SSA, anti-SSB, age, gender, C3, C4, dry eyes, or dry mouth between the two groups.

**Table 2:** Clinical characteristics of patients with pSS-EGM and pSS-non-EGM

Characteristics	pSS-EGM	pSS-non-EGM	*P*-value
N	50	13	
Age, years, mean (range)	52.84 ± 13.49	45.77 ± 13.31	0.105
Female, n (%)	43/50 (86.00)	12/13 (92.31)	0.888
Anti-SSA-positive (%)	39/50 (78.00)	10/13 (76.92)	1
Anti-SSB-positive (%)	25/50 (50.00)	3/13 (23.08)	0.154
ANA-positive (%)	37/50 (74.00)	5/13 (38.46)	0.036
RF-positive (%)	16/41 (39.02)	0/12 (0.00)	0.026
Duration(month)	32.39 ± 47.14	28.15 ± 64.92	0.156
CRP (8 mg/L)	8.46 ± 19.57	2.49 ± 5.42	0.542
ESR (mm/h)	37.93 ± 21.06	11.69 ± 7.30	< 0.001
IgG (g/L)	23.09 ± 8.87	14.72 ± 1.96	< 0.001
IgA (g/L)	3.84 ± 1.55	3.24 ± 0.85	0.124
IgM (g/L)	1.51 ± 0.79	1.29 ± 0.57	0.355
C3 (g/L)	1.04 ± 0.21	1.06 ± 0.18	0.751
C4 (g/L)	0.20 ± 0.08	0.24 ± 0.09	0.230
FS ≥ 1	39/50 (78.00)	11/13 (84.62)	0.888
ESSDAI	14.52 ± 10.16	1.08 ± 2.02	< 0.001
Xerophthalmia (%)	27/50 (54.00)	5/13 (38.46)	0.492
Xerostomia (%)	26/50 (52.00)	6/13 (46.15)	0.949

Data are expressed as mean ± SD or n (%)

*ANA* Antinuclear Antibody, *C3* Complement C3, *C4* Complement C4, *CRP* C-reaction protein, *ESR* Erythrocyte Sedimentation Rate, *ESSDAI* EULAR Sjögren's Syndrome Disease Activity Index, *FS* Focus score, *IgA* Immunoglobulin A, *IgG* Immunoglobulin G, *IgM* Immunoglobulin M, *RF* Rheumatoid Factor, *SSA* Anti-SSA antibody, *SSB* Anti-SSB antibody

### Detecting DEGs in pSS patients

**Fig. 1:**
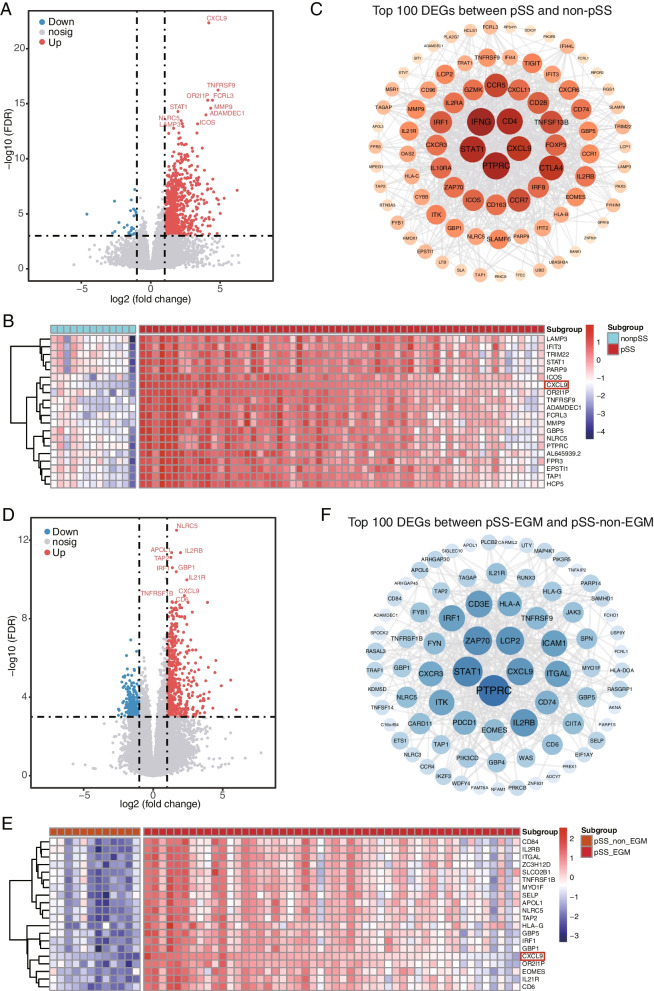
Identification of DEGs in patients. **A** Volcano plot representing differentially expressed genes between pSS and non-pSS samples. Genes with adjusted *p* < 0.001 and absolute log2-fold change > 1 are represented in red or blue. **B** Heatmap showing the top 20 DEGs between pSS and non-pSS. **C** The network consists of 100 top DEGs between pSS and non-pSS. **D** Volcano plot representing differentially expressed genes between pSS-EGM and pSS-non-EGM samples. Genes with adjusted *p* < 0.001 and absolute log2-fold change > 1 are represented in red or blue. **E** Heatmap showing the top 20 DEGs between pSS-EGM and pSS-non-EGM. **F** The network consists of 100 top DEGs between pSS-EGM and pSS-non-EGM. In the network, each gene is represented by a node, and the connections between nodes represent the interactions between the corresponding proteins. The size and color of the nodes indicate the degree (number of connection) of the genes. The larger size and darker color indicate a higher degree and greater importance

Using RNA sequencing, we analyzed gene expression patterns of MSGs in 63 pSS patients and 12 non-pSS patients. We discovered 725 genes that exhibit varying levels of expression between individuals with pSS and non-pSS subjects, with 697 of these genes (96.1%) being up-regulated. Among these DEGs, *CXCL9* showed the most significant difference in expression (Fig. 1A). Figure 1B displays the top 20 DEGs. In addition, we used Cytoscape v3.9.0 software to construct a PPI network graph (Fig. 1C), which revealed hub genes at the central position, including *STAT1*, *IFNG*, *CD4*, *CXCL9*, *PTPRC*.

Furthermore, we used RNA sequencing to analyze the gene expression patterns in patients with pSS, both with and without EGMs. We found 727 genes that were expressed differently between the two groups, with 517 (71.1%) of these genes being up-regulated. Notably, *CXCL9* had significantly higher expression levels in pSS patients with EGMs than in those without, as shown in Fig. 1D. The top 20 DEGs, including *CD84*, *IRF1*, *IL21RB*, *CXCL9*, and others, are displayed in Fig. 1E. Additionally, the hub genes at the central position include *STAT1*, *ZAP70*, *LCP2*, *CXCL9*, *PTPRC* (Fig. 1F).

### Pathway enrichment analysis of upregulated DEGs

**Fig. 2:**
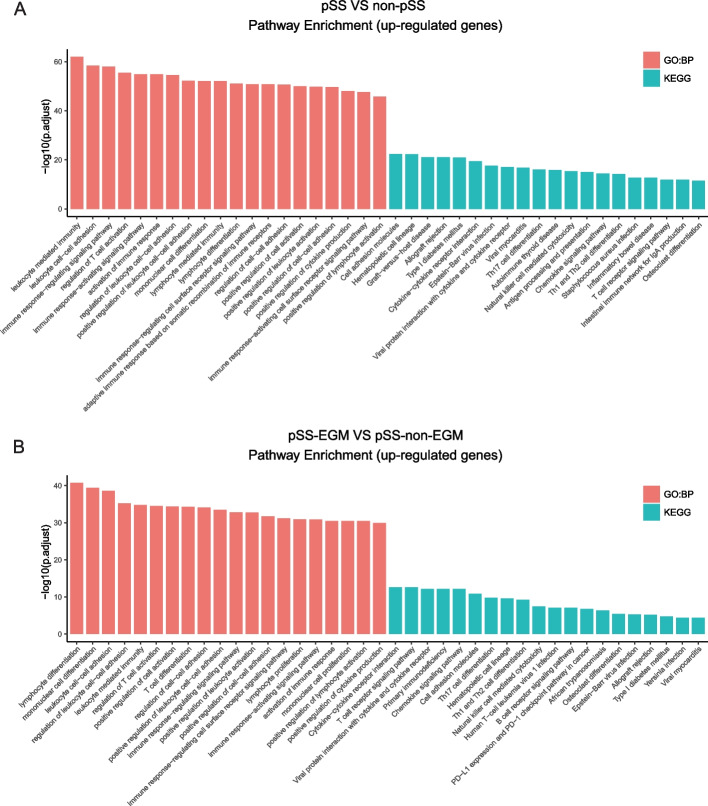
Pathway enrichment analysis of upregulated DEGs. **A** Bar chart depicting the top 20 enriched Gene Ontology (GO) and KEGG pathways from the analysis of upregulated DEGs between pSS and non-pSS. **B** Bar chart illustrating the top 20 enriched Gene Ontology (GO) and KEGG pathways from the analysis of upregulated DEGs between pSS-EGM and pSS-non-EGM. The x-axis displays the GO and KEGG pathways, while the y-axis shows the enrichment score (-log10 adjusted *p*-value). Each bar corresponds to a specific pathway, with its height representing the degree of enrichment

In order to investigate potential biological mechanisms associated with pSS and extra-glandular involvement in pSS, we conducted analyses on commonly upregulated DEGs using GO and KEGG pathway methods. In Fig. 2A, the analysis of upregulated DEGs between pSS and non-pSS reveals the top 20 pathways that are enriched. In pSS, several immune-related pathways showed significant upregulation, including leukocyte cellular adhesion, differentiation of T cells, and proliferation of lymphocytes in GO. Additionally, there was an upregulation in pathways such as Cytokine-cytokine receptor interaction, T cell receptor signaling, and Chemokine signaling in KEGG.

Moreover, Fig. 2B demonstrates the top 20 enriched GO and KEGG pathways resulting from the examination of upregulated DEGs between pSS-EGM and pSS-non-EGM. In a similar manner as the previous outcome, a significant number of immune-related pathways exhibit upregulation in pSS-EGM in comparison to pSS-non-EGM. Additionally, the Chemokine signaling pathway is an important pathway in both groups.

### Clinical correlations of *CXCL9* in pSS

**Fig. 3:**
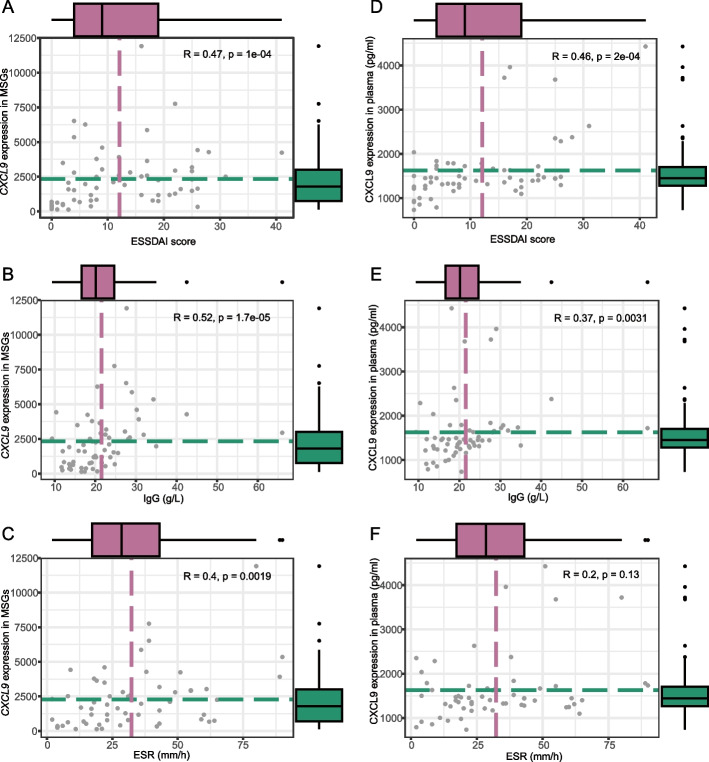
Association between CXCL9 and clinical features of primary Sjögren's syndrome. **A**, **B**, **C** The Spearman correlation between expression of *CXCL9* gene in MSG and clinical characteristics such as ESSDAI score (**A**), IgG (**B**), and ESR (**C**). **D**, **E**, **F** The Spearman correlation between expression of *CXCL9* gene in plasma and clinical characteristics such as ESSDAI score (**D**) and IgG (**E**), but not with ESR (**F**). The top and right boxplot indicates the distribution. Dashed lines represent the mean value

To determine how potential biomarkers affect the severity of pSS-EGM disease, we analyzed the correlation between the top 20 DEGs (DEGs between pSS-EGM and pSS-non-EGM) and various clinical parameters, including ESSDAI scores, ESR, CRP, IgA, IgG, IgM, and other clinical parameters. Among the 20 genes, only *CXCL9* and *PTPRC* were identified as the central hub genes. Moreover, it was observed that *CXCL9* exhibited a stronger correlation with the clinical parameters. Therefore, we have selected CXCL9 for additional analysis and included it in the Supplementary materials. Our analysis showed that *CXCL9* in MSG had a positive correlation with ESSDAI score (Fig. 3A, *r* = 0.47,* p* < 0.001), IgG levels (Fig. 3B, *r* = 0.52, *p* < 0.001), and ESR (Fig. 3C, *r* = 0.40, *p* = 0.002). Nevertheless, no significant associations were found between *CXCL9* RNA expression and other clinical factors.

### Assessment of circulating chemokine CXCL9 and CXCR3

**Fig. 4:**
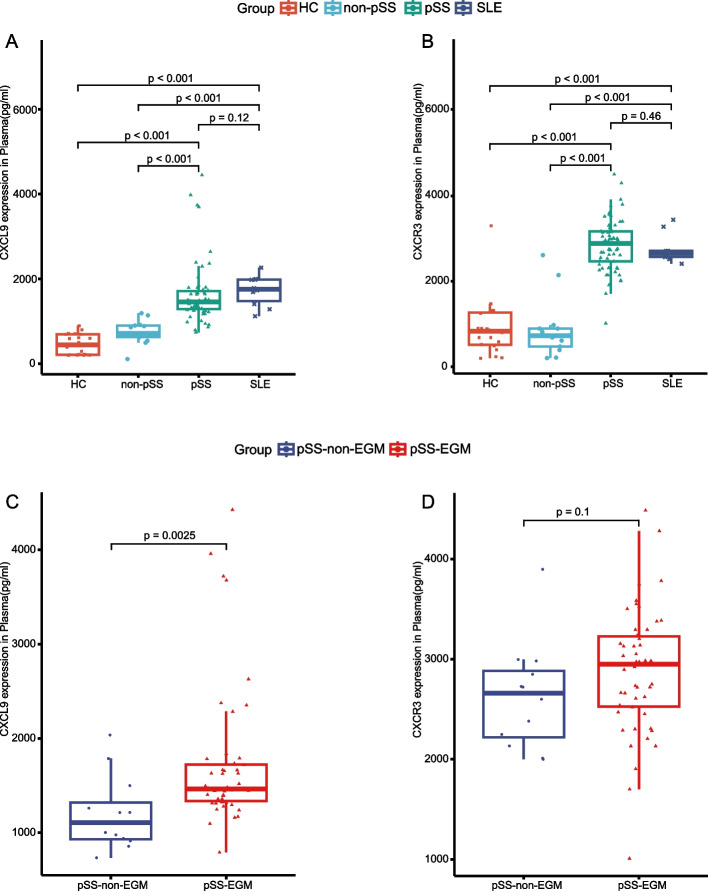
Differential expression of CXCL9 and CXCR3 in plasma. **A**, **B** Boxplots depicting the expression of plasma CXCL9 (**A**) and CXCR3 (**B**) in HC, non-pSS, pSS, SLE. **C**, **D** Boxplots showing the expression of plasma CXCL9 (**C**) and CXCR3 (**D**) between pSS-non-EGM and pSS-EGM. Significance determined by Wilcoxon's test

ELISA was used to measure CXCL9 and CXCR3 levels in plasma from patients with pSS, non-pSS, as well as 10 SLE patients and 20 healthy individuals. A higher level of CXCL9 and CXCR3 was noted in both pSS and SLE patients (Fig. 4A, B). Furthermore, a notably increase in CXCL9 expression was found in plasma of pSS patients with EGM. In contrast, CXCR3 expression did not display significant differences between pSS patients with and without EGM (Fig. 4C, D).

Furthermore, a strong association was observed between CXCL9 levels in plasma and both ESSDAI score and IgG levels. The correlation coefficients were 0.46 (*p* < 0.001) and 0.37 (*p* = 0.003), respectively (Fig. 3D, E, F).

### *CXCL9* expression in pSS with EGM

**Fig. 5:**
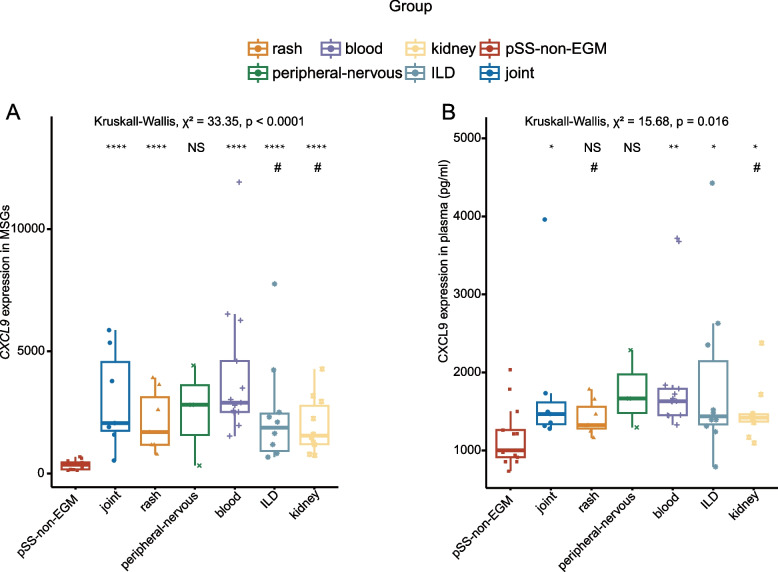
Differentially expressed *CXCL9* in pSS subgroups with different EGM. **A** Box plot showing the expression of *CXCL9* in pSS-EGM subgroups and pSS-non-EGM in MSGs. **B** Box plot showing the expression of CXCL9 in pSS-EGM subgroups and pSS-non-EGM in plasma. Significance determined by Kruskall-Wallis’s test. The “*” symbol denotes the significance between pSS-non-EGM and pSS-EGM subgroups. The “#” symbol denotes the significance between blood involvement and other subgroups within the pSS-EGM group. ^*^ *p* < 0.05, ^**^p < 0.01, ^***^ p < 0.001, ^****^ p < 0.001; ^#^* p* < 0.05, ^##^* p* < 0.01, ^###^* p* < 0.001, ^####^* p* < 0.001

The study measured the expression of *CXCL9* in MSGs and blood plasma of pSS patients with EGM. We sorted the patients into six subgroups based on their symptoms: peripheral blood involvement, joint involvement, kidney involvement, ILD, peripheral nervous system involvement, and rash involvement. There was a notable rise in *CXCL9* expression in patients with joint involvement, rash involvement, blood involvement, kidney involvement, and ILD, compared to pSS-non-EGM patients, except for those with peripheral nervous system involvement. Furthermore, patients with blood involvement exhibited higher *CXCL9* expression levels than patients with kidney involvement and ILD (Fig. 5A). Similarly, CXCL9 expression in plasma was significantly higher in patients with joint involvement, blood involvement, kidney involvement, and ILD, compared to pSS-non-EGM patients, except for those with peripheral nervous system involvement and rash involvement. Moreover, pSS patients with blood involvement had the highest levels of CXCL9 expression (Fig. 5B).

### *CXCL9* expression is elevated in pSS with SSA antibodies

The study discovered that pSS patients who have SSA antibodies may have higher levels of *CXCL9* expression in their minor salivary gland (MSG) and plasma. Antibodies against SSA were used to classify patients. A notable distinction was found between patients with antibodies and those without antibodies in the expression of the *CXCL9* gene in MSG and the plasma levels of the protein, as shown in Figure S2A and S2B.

## Discussion

Although there is a wealth of literature on the application of RNA-seq in investigating pSS patients [36–40], research on pSS patients with EGM is relatively limited. For this research, we employed RNA-seq to examine the gene expression patterns of diagnosed pSS patients in comparison to individuals without pSS, as well as pSS patients with and without EGM. In pSS, there are disruptions in several immune-related pathways, and these disruptions also occur in cases of pSS with extra-glandular involvement. Among these pathways, the Chemokine signaling pathway was found to be particularly important in both groups, which is consistent with prior studies on pSS [41, 42]. Additionally, among several DEGs, *CXCL9* showed a significant difference in expression in pSS. Furthermore, *CXCL9* has the potential to act as a critical regulator or signaling molecule in the network that drives the progression of pSS and pSS with EGM.

CXCL9 is a chemokine protein that interacts with its receptor, CXCR3, to attract T cells, natural killer cells, and macrophages to the location of inflammation [43–45]. Interferon-gamma-induced CXCL9 is also called MIG (monokine induced by interferon gamma) and is believed to be involved in the development of multiple autoimmune disease [46–48], including pSS [23, 49]. Several studies [13, 50] have reported elevated levels of *CXCL9* in the exocrine glands tissue of patients with pSS, and our results were consistent with what has been previously described in the literature. However, the correlation between CXCL9 and extra-glandular involvement of pSS has not been previously investigated.Our study found that the expression of *CXCL9* is higher in MSGs from pSS patients compared to non-pSS subjects. Furthermore, it was observed that pSS and SLE patients had elevated levels of CXCL9 and CXCR3 in the plasma when compared to individuals of non-pSS and healthy controls. Numerous studies [51–53] have suggested that the development of pSS and SLE shares some similarities. Our findings indicate that the CXCL9/CXCR3 axis may have a significant role in the pathogenesis of both pSS and SLE. Moreover, we discovered a connection between the *CXCL9* gene's expression and CXCL9 protein levels, as well as various clinical characteristics in these individuals, including ESR, IgG levels, and ESSDAI. This suggests that biomarker *CXCL9* may be potentially serve as a helpful indicator for assessing both disease activity and progression in individuals with pSS. Such information could assist clinicians in making more informed decisions regarding treatment options for their patients, indicating that *CXCL9* could play a role in the progression of pSS.

Our findings indicate that compared to patients without EGM, patients with pSS who exhibit EGM have higher levels of ANA, RF, ESR, IgG, and ESSDAI (all *p*-values < 0.05). Due to the importance of these clinical parameters in the diagnosis and treatment of pSS [54–57], it is essential to consider them when evaluating patients with confirmed pSS. This will facilitate early detection of EGM in pSS patients. Additionally, we found a considerable rise in the expression of the *CXCL9* gene and CXCL9 protein levels in pSS patients with EGM compared to those without EGM. It is known that the *CXCL9/CXCR3* pathway is critical in the migration of immune cells [43, 58, 59]. Studies have shown that the migration of a large number of immune cells and invasion of glandular tissue are the first and most important pathological manifestations of pSS [13, 49, 56]. This suggests that the occurrence of EGM in pSS patients could be related to the *CXCL9/CXCR3* pathway. There are various manifestations of extra-glandular involvement in patients with pSS [60, 61]. To further understand of the role of CXCL9 in various types of extra-glandular involvement, the pSS-EGM group was divided into six subgroups. In addition to peripheral nervous system and rash involvement, the expression of CXCL9 was noticeably higher in pSS patients with joint, blood, kidney, and ILD involvement in comparison to pSS-non-EGM patients. Patients with blood involvement showed the highest levels of CXCL9 expression. Further research is required to explore the relationship between EGM and the CXCL9/CXCR3 pathway.

The anti-SSA antibody, also known as anti-Ro antibody, is an autoantibody that targets the SSA antigen [62, 63]. It is one of the diagnostic criteria for the disease [28, 55]. Previous studies [6, 64, 65] have found that the existence of SSA antibodies is a reliable indication of the existence of EGM in pSS over an extended period of observation. However, our study discovered that the rates of positivity in SSA showed no significant variation among patients with and without EGM. The limited number of pSS patients in our sample may have influenced the research findings, and race differences may have also played a role. To verify these findings in the future, it may be necessary to conduct an ongoing follow-up study. Additionally, our research revealed that pSS patients with SSA antibodies have elevated levels of *CXCL9* expression in their MSG and higher levels of CXCL9 protein in their plasma. Earlier studies [66, 67] have shown that anti-SSA antibodies can trigger activated interferon (IFN) production by activating plasmacytoid dendritic cells, which are specialized immune cells that produce significant quantities of IFN. This can in turn result in the production of CXCL9 via MIG signaling. Therefore, there may be a correlation between anti-SSA in pSS and CXCL9 mediated by IFN signaling. Nevertheless, further investigation is necessary to clarify the precise mechanisms and implications of this connection.

## Conclusions

Overall, these findings confirm that *CXCL9* is elevated in pSS patients, particularly those with EGM and SSA antibodies. The way it is expressed may be a useful biomarker for measuring disease severity, suggesting that it could have a significant impact on the development of the disease. It could also be utilized as a biomarker or treatment target, but additional research is necessary to validate and fully understand these discoveries.

### Supplementary Information

**Additional file 1: Table S1.** Clinical characteristics of patients with pSS and SLE.

**Additional file 2: Table S2.** The correlation between top 20 DEGs and clinical features.

**Additional file 3: Table S3.** The results of the Shapiro–Wilk test.

**Additional file 4: Figure S1.** Association Between CXCL9 and ClinESSDAI score of Primary Sjögren's Syndrome. (A) The Spearman correlation between expression of CXCL9 gene in MSG and ClinESSDAI score. (B) The Spearman correlation between expression of CXCL9 gene in plasma and ClinESSDAI score. The top and right boxplot indicates the distribution. Dashed lines represent the mean value.

**Additional file 5: Figure S2.** Differentially expressed CXCL9 between pSS patients with or without SSA antibodies. (A) Box plot illustrates the CXCL9 expression in MSGs among pSS patients with or without SSA antibodies. (B) Box plot showing the expression of CXCL9 between pSS patients with or without SSA antibodies in plasma. Significance determined by Wilcoxon's test.

## Data Availability

Data used during the study can be obtained from a reasonable request to the corresponding author.

## Abbreviations

ANA: Antinuclear antibody
CXCL9: C-X-C motif chemokine ligand 9
CXCR3: C-X-C motif chemokine receptor 3
DEGs: Differentially expressed genes
EGM: Extra-glandular manifestations
ESR: Erythrocyte sedimentation rate
ESSDAI: EULAR Sjogren’s Syndrome Disease Activity Index
IgG: Immunoglobulin G
ILD: Interstitial lung disease
MSG: Minor salivary gland
pSS: Primary Sjögren's syndrome
SLE: Systemic lupus erythematosus
