# Supplementary material for: CXCL9 may serve as a potential biomarker for primary Sjögren’s syndrome with extra-glandular manifestations
Source: Arthritis Res Ther. 2024 Jan 17;26:26. doi: 10.1186/s13075-023-03229-x (PMC10792874; doi:10.1186/s13075-023-03229-x)
Supplement: Supplementary file 1 — Additional file 1: Table S1. Clinical characteristics of patients with pSS and SLE. [file 13075_2023_3229_MOESM1_ESM.docx]

Table S1. Clinical characteristics of patients with pSS and SLE

| Characteristics | pSS | SLE | *P*-value |
| --- | --- | --- | --- |
| N | 63 | 10 |  |
| Age, years, mean (range) | 50.85 ± 15.90 | 47.66 ± 16.03 | 0.342 |
| Female, n (%) | 55/63 (87.30) | 9/10 (90.00) | 1 |
| ANA-positive (%) | 42/63(66.67) | 10/10 (100) | <0.001 |
| Anti-SSA-positive (%) | 49/63(77.78) | 6/10 (60) | 0.146 |
| Anti-SSB-positive (%) | 28/63 (44.44) | 4/10 (40) | 0.542 |

Data are expressed as mean ± SD or n (%).

ANA, Antinuclear Antibody; SSA, Anti-SSA antibody; SSB, Anti-SSB antibody.
