# Supplementary figures and images for: CXCL9 may serve as a potential biomarker for primary Sjögren’s syndrome with extra-glandular manifestations
Source: Arthritis Res Ther. 2024 Jan 17;26:26. doi: 10.1186/s13075-023-03229-x (PMC10792874; doi:10.1186/s13075-023-03229-x)

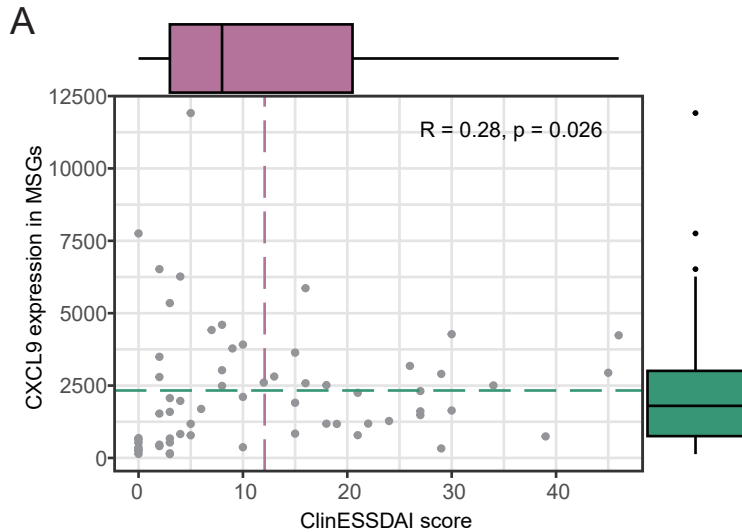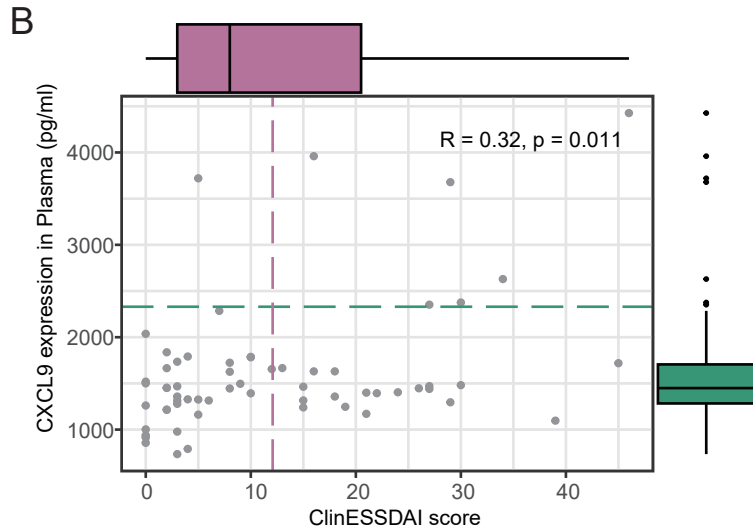

Supplement: Supplementary file 4 — Additional file 4: Figure S1. Association Between CXCL9 and ClinESSDAI score of Primary Sjögren's Syndrome. (A) The Spearman correlation between expression of CXCL9 gene in MSG and ClinESSDAI score. (B) The Spearman correlation between expression of CXCL9 gene in plasma and ClinESSDAI score. The top and right boxplot indicates the distribution. Dashed lines represent the mean value. [file 13075_2023_3229_MOESM4_ESM.pdf]

Group 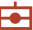 SSA+ 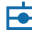 SSA-

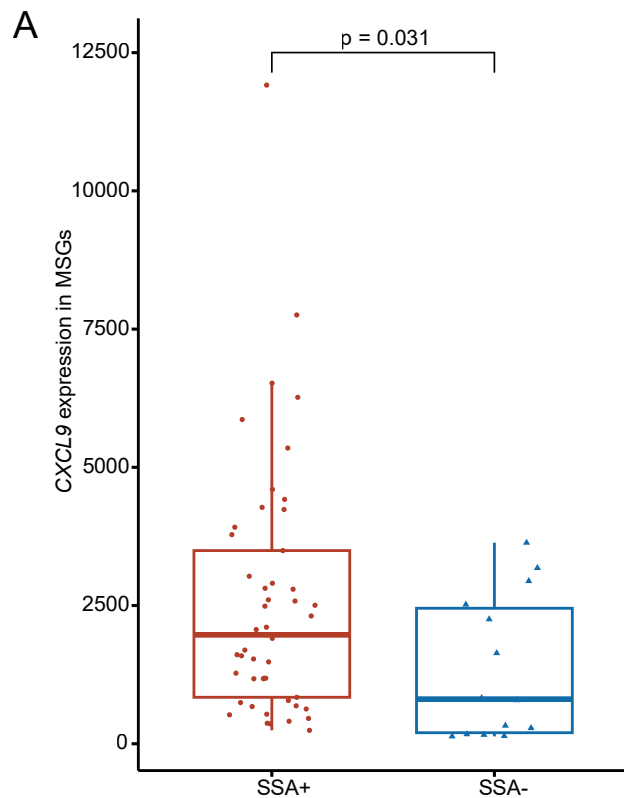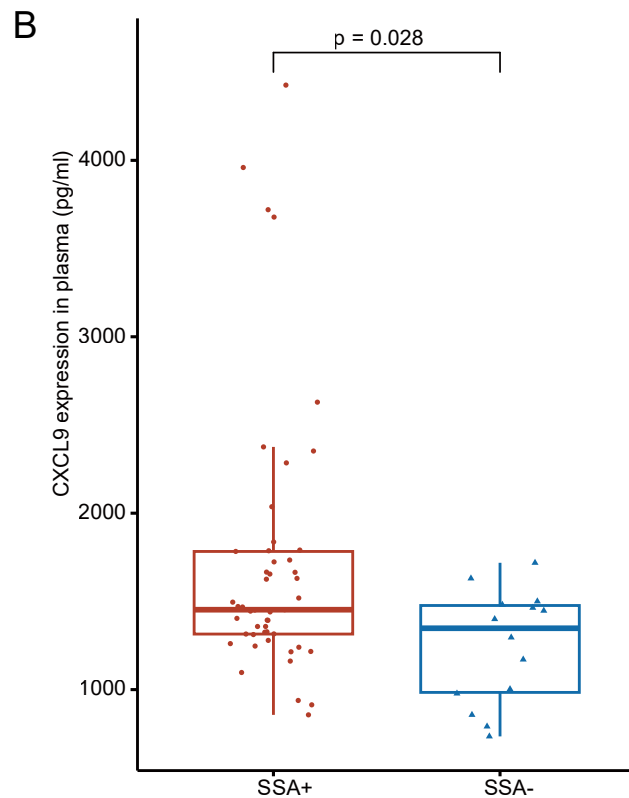

Supplement: Supplementary file 5 — Additional file 5: Figure S2. Differentially expressed CXCL9 between pSS patients with or without SSA antibodies. (A) Box plot illustrates the CXCL9 expression in MSGs among pSS patients with or without SSA antibodies. (B) Box plot showing the expression of CXCL9 between pSS patients with or without SSA antibodies in plasma. Significance determined by Wilcoxon's test. [file 13075_2023_3229_MOESM5_ESM.pdf]
